# Retraction: Prognostic influence of PD-1/PD-L1 suppressors in combination with chemotherapeutic agents for non-small cell pulmonary carcinoma: system review and meta-analysis

**DOI:** 10.3389/fonc.2024.1383366

**Published:** 2024-02-19

**Authors:** Frontiers Editorial Office

## Introduction

The journal retracts the 2023 article cited above.

Following publication, the publisher identified concerns regarding the scope and peer review of this article. A subsequent investigation, which was conducted in accordance with Frontiers’ policies, found indicators of third-party involvement and inadequate peer review process. As the scientific integrity of the article cannot be guaranteed, and adhering to the recommendations of the Committee on Publication Ethics (COPE), the article is retracted.

The authors have not responded to this retraction.

This retraction was approved by the Chief Editors of Frontiers in Oncology and the Chief Executive Editor of Frontiers.

